# Supplementary material for: Hyper-mutational processes provide a head-start for weak cancer drivers: Explaining atypical KRAS variants
Source: PLoS Comput Biol. 2026 Oct 9;22(10):e1014858. doi: 10.1371/journal.pcbi.1014858 (PMC13674863; doi:10.1371/journal.pcbi.1014858)
Supplement: Supplementary file 2 [file pcbi.1014858.s005.pdf]

Study	Samples
**cBioPortal: bowel_colitis_msk_2022**	89
**cBioPortal: coad_caseccc_2015**	29
**cBioPortal: coad_cptac_2019**	110
**cBioPortal: coadread_dfci_2016**	619
**cBioPortal: coadread_genentech**	74
**cBioPortal: coadread_mskcc**	138
**cBioPortal: coadread_mskresistance_2022**	22
**cBioPortal: coadread_tcga**	640
**cBioPortal: coadread_tcga_pan_can_atlas_2018**	594
**cBioPortal: coadread_tcga_pub**	276
**cBioPortal: crc_apc_impact_2020**	471
**cBioPortal: crc_dd_2022**	47
**cBioPortal: crc_msk_2017**	1134
**cBioPortal: crc_nigerian_2020**	64
**cBioPortal: rectal_msk_2019**	339
**cBioPortal: rectal_msk_2022**	801
**100,000 genomes project V8 (only with oncogenic KRAS)**	816

Supplementary table 1: *KRAS* mutation data from colorectal cancer samples provided by the listed studies were downloaded from the cBioPortal data portal and from the Genomics England Research Environment.
