# Supplementary material for: Hyper-mutational processes provide a head-start for weak cancer drivers: Explaining atypical KRAS variants
Source: PLoS Comput Biol. 2026 Oct 9;22(10):e1014858. doi: 10.1371/journal.pcbi.1014858 (PMC13674863; doi:10.1371/journal.pcbi.1014858)
Supplement: Supplementary file 3 [file pcbi.1014858.s006.pdf]

mutCds	mutAA	triContext	mutType	boostDM	codon	relMutRateCrcPole	relMutRateCrc	relMutRateMSI	haigisActivationScore	classActScore	class1213	classCbioCnt	classCbioCntMssStrict
**c.35G>A**	p.G12D	ACC	A[C>T]C	TRUE	12	3.62239079403612E-11	1.26539687391834E-10	2.00873845495349E-10	5130	Strong	Strong	Strong	Strong
**c.35G>T**	p.G12V	ACC	A[C>A]C	TRUE	12	4.43596182839995E-11	1.69481699622471E-10	5.35602218848631E-11	1726.2	Weak	Strong	Strong	Strong
**c.34G>T**	p.G12C	CCA	C[C>A]A	TRUE	12	2.13062918431402E-11	1.71464299371741E-10	6.22217428590003E-11	3283	Strong	Strong	Weak	Weak
**c.35G>C**	p.G12A	ACC	A[C>G]C	TRUE	12	1.47299056343287E-12	4.9765478190354E-11	3.50648720519989E-11	165.1	Weak	Strong	Strong	Weak
**c.34G>A**	p.G12S	CCA	C[C>T]A	TRUE	12	1.18160795390179E-12	1.055209390569E-10	9.10190160060388E-11	NA	Weak	Strong	Weak	Weak
**c.34G>C**	p.G12R	CCA	C[C>G]A	TRUE	12	3.16113917279636E-13	2.85774878890389E-11	2.23179653509995E-11	626.4	Weak	Strong	Weak	Weak
**c.38G>A**	p.G13D	GCC	G[C>T]C	TRUE	13	1.42844955650311E-10	1.73930360392434E-10	6.37296093474457E-10	1238.4	Weak	Strong	Strong	Weak
**c.37G>T**	p.G13C	CCA	C[C>A]A	TRUE	13	2.13062918431402E-11	1.71464299371741E-10	6.22217428590003E-11	NA	Weak	Strong	Weak	Weak
**c.37G>C**	p.G13R	CCA	C[C>G]A	FALSE	13	3.16113917279636E-13	2.85774878890389E-11	2.23179653509995E-11	NA	Weak	Strong	Weak	Weak
**c.38G>C**	p.G13A	GCC	G[C>G]C	FALSE	13	1.05727839818753E-12	4.22082807957858E-11	3.38044487282723E-11	NA	Weak	Strong	Weak	Weak
**c.40G>A**	p.V14I	ACG	A[C>T]G	TRUE	14	3.15746280783741E-10	4.51795635394046E-09	3.97129778398875E-09	NA	Weak	Weak	Weak	Weak
**c.57G>T**	p.L19F	TCA	T[C>A]A	TRUE	19	4.56677959865201E-10	2.41119142293691E-10	4.63834359092911E-11	NA	Weak	Weak	Weak	Weak
**c.57G>C**	p.L19F	TCA	T[C>G]A	TRUE	19	5.0656627325839E-13	6.7650379355931E-11	2.63649771456482E-11	NA	Weak	Weak	Weak	Weak
**c.64C>A**	p.Q22K	ACA	A[C>A]A	FALSE	22	1.16251920625484E-11	1.93235767894909E-10	4.25048801663535E-11	NA	Weak	Weak	Weak	Weak
**c.99T>G**	p.D33E	ATC	A[T>G]C	FALSE	33	6.50099468592995E-11	1.7950428131891E-11	1.4807468546693E-11	NA	Weak	Weak	Weak	Weak
**c.99T>A**	p.D33E	ATC	A[T>A]C	FALSE	33	3.78235366757319E-12	5.54923094650923E-11	5.26132346504455E-11	NA	Weak	Weak	Weak	Weak
**c.101C>T**	p.P34L	CCA	C[C>T]A	TRUE	34	1.18160795390179E-12	1.055209390569E-10	9.10190160060388E-11	NA	Weak	Weak	Weak	Weak
**c.101C>G**	p.P34R	CCA	C[C>G]A	TRUE	34	3.16113917279636E-13	2.85774878890389E-11	2.23179653509995E-11	NA	Weak	Weak	Weak	Weak
**c.169G>A**	p.D57N	TCG	T[C>T]G	FALSE	57	7.30979868909882E-09	2.28019549233063E-09	1.61670356516155E-09	NA	Weak	Weak	Weak	Weak
**c.176C>A**	p.A59E	GCA	G[C>A]A	TRUE	59	9.80561990675538E-12	3.22514505165099E-10	4.62257172448083E-11	NA	Weak	Weak	Weak	Weak
**c.175G>A**	p.A59T	GCT	G[C>T]T	FALSE	59	1.71323141220487E-10	1.4447089175461E-10	4.15453169809831E-10	NA	Weak	Weak	Weak	Weak
**c.179G>A**	p.G60D	ACC	A[C>T]C	TRUE	60	3.62239079403612E-11	1.26539687391834E-10	2.00873845495349E-10	NA	Weak	Weak	Weak	Weak
**c.183A>C**	p.Q61H	CTT	C[T>G]T	TRUE	61	2.36329082944057E-10	1.65846775458783E-10	3.12169596928711E-11	NA	Weak	Weak	Weak	Weak
**c.183A>T**	p.Q61H	CTT	C[T>A]T	TRUE	61	4.34976438948716E-12	8.11138889786151E-11	1.98036751446009E-11	NA	Weak	Weak	Weak	Weak
**c.182A>T**	p.Q61L	TTG	T[T>A]G	TRUE	61	4.10154613547327E-12	2.71697103842738E-11	1.71392295651026E-11	107.2	Weak	Weak	Weak	Weak
**c.182A>G**	p.Q61R	TTG	T[T>C]G	TRUE	61	6.22959836643168E-12	4.7624775213001E-11	1.12951694300191E-10	NA	Weak	Weak	Weak	Weak
**c.182A>C**	p.Q61P	TTG	T[T>G]G	TRUE	61	3.76567362332351E-11	4.00128387629334E-11	2.88797059071623E-11	NA	Weak	Weak	Weak	Weak
**c.181C>G**	p.Q61E	TCA	T[C>G]A	TRUE	61	5.0656627325839E-13	6.7650379355931E-11	2.63649771456482E-11	NA	Weak	Weak	Weak	Weak
**c.181C>A**	p.Q61K	TCA	T[C>A]A	TRUE	61	4.56677959865201E-10	2.41119142293691E-10	4.63834359092911E-11	NA	Weak	Weak	Weak	Weak
**c.351A>T**	p.K117N	ATT	A[T>A]T	TRUE	117	3.10376418373657E-11	1.02282891845063E-10	8.9341534471008E-11	NA	Weak	Weak	Weak	Weak
**c.350A>G**	p.K117R	TTT	T[T>C]T	FALSE	117	9.85601525806229E-12	5.77589170440001E-11	1.59018983932647E-10	NA	Weak	Weak	Weak	Weak
**c.351A>C**	p.K117N	ATT	A[T>G]T	FALSE	117	2.35884837058572E-10	4.40035902794003E-11	2.1874263276468E-11	NA	Weak	Weak	Weak	Weak
**c.436G>A**	p.A146T	GCT	G[C>T]T	TRUE	146	1.71323141220487E-10	1.4447089175461E-10	4.15453169809831E-10	NA	Weak	Weak	Weak	Weak
**c.437C>T**	p.A146V	GCA	G[C>T]A	TRUE	146	1.10818917528406E-11	1.33101336610765E-10	5.10048895384325E-10	NA	Weak	Weak	Weak	Weak
**c.436G>C**	p.A146P	GCT	G[C>G]T	FALSE	146	5.26495896343364E-13	4.00916029735082E-11	3.5131634434728E-11	NA	Weak	Weak	Weak	Weak
**c.439A>G**	p.K147E	TTT	T[T>C]T	FALSE	147	9.85601525806229E-12	5.77589170440001E-11	1.59018983932647E-10	NA	Weak	Weak	Weak	Weak

Supplementary table 2: *KRAS* driver variants considered in this study and their classifications as weak or strong drivers based on criteria as explained in supplementary information.
