# Supplementary material for: Hyper-mutational processes provide a head-start for weak cancer drivers: Explaining atypical KRAS variants
Source: PLoS Comput Biol. 2026 Oct 9;22(10):e1014858. doi: 10.1371/journal.pcbi.1014858 (PMC13674863; doi:10.1371/journal.pcbi.1014858)
Supplement: Supplementary file 4 [file pcbi.1014858.s009.pdf]

**Dr Michael Nicholson,**
**School of Mathematics and**
**The Institute for Genetics and Cancer,**
**University of Edinburgh**
**Edinburgh, EH9 3FD. UK**

**Professor Ian Tomlinson FRS FMedSci,**
**Department of Oncology,**
**University of Oxford**
**Oxford, OX3 7DQ. UK**

20h March 2026

Dear Professor Bozic,

Thank you for your continued consideration of our manuscript. We are pleased to see that Reviewer 2 is satisfied with the paper.

In your most recent message you communicated that the concerns of Reviewer 1 need to be addressed. This reviewer is clearly someone who is experienced in the field, but their comments and overall attitude are most unusual, and indeed represent a unique outlier in the nearly 1000 manuscripts published by the co-authors of the present study.

Specifically,

1) The reviewer suggested 15 references be added in their initial review. We carefully evaluated each of these references, relating them to the sentence for which the reviewer suggested them. We found that many references were not relevant to the point being made, and in fact some distracted from the point. We also noted a concentration of suggested references from one particular group, which we believe is concerning. Whilst it is entirely acceptable for a scientist to hold strong beliefs about the virtues of their work, it is not appropriate for them to, in effect, hold the authors of papers to ransom, by making acceptance of a paper conditional on adding multiple citations to their work. We are happy to provide a paper-by-paper criticism of the suggested additional references, although we believe that we appropriately reference the literature. A further 9 references were cited when requesting additional analysis be performed, also with the same concentration from one group.

2) The reviewer is unsatisfied with our model parameterisation. In their original review, the reviewer claimed our model was “parameterized arbitrarily”. In actuality, our mutation rate estimates were parameterised with recent genomic data and our selection parameters are drawn from recent publications using the same modelling framework. Therefore we highlighted this to the reviewer.

   Now the reviewer appears to desire that we use software from the Townsend group to parameterise our model (this methodology was suggested by the reviewer in the first review round, no approach was specifically suggested in their most recent message). However, the selection coefficients obtained from the Townsend group’s methodology

assume a Moran-type process model of mutation fixation in a fixed size tumour (as can be seen in the supplementary information of the original publication PMID: 30365005), while we focus on secondary mutations in a branching process model. Despite the reviewer asserting that it is possible, you yourself have previously demonstrated (PMID: 34983781) that selection coefficients from one model cannot routinely or straightforwardly be transferred or adapted to another model. We are happy to provide an extensive commentary on this point, but do not believe this will satisfy the reviewer. We are unwilling to completely redesign our methodological approach, an approach which is standard within the cancer modelling community and scientifically sound.

Our conclusions are based on a broad range of selection parameter values, that range being based on recent publications that used the same modelling framework as us (including several of your own). If our parameter range is not empirically justified as the reviewer claims, these recent publications that infer selection coefficients must also be deficient, although the reviewer does not claim this or explain their reasoning. Our estimates of mutation rates are standard in the field and use recently available mutational signature data.

3) The reviewer asks for epistasis to be included in the model, as they claim it is an alternative explanatory mechanism. However, they make no clear argument as to why it is an alternative explanatory mechanism, nor how they would like the analysis to be amended to incorporate the effect. We have explained that we accept (and have in the past even argued for) epistasis as a possible cause of the pervasiveness of weaker driver genes, but that this is not the focus of our study.

   From a modelling perspective, in our previous response we explained that antagonistic epistasis will have little effect in our model as strong driver sweeps arise from the population without the weak driver mutation - the reviewer did not engage with that argument. If we amend our model to include cells with both weak and strong drivers, these will arrive latest in the model and will grow slowly with antagonistic selection - thus there will be no effect. We are happy to provide simulations on this point.

   From a data perspective, we added a discussion of a recently published co-occurrence analysis of KRAS variants, which directly addresses the question the reviewer raised and supports our modelling assumptions. The reviewer appears unsatisfied with the methodology of that study. They are entitled to that opinion, but asking us to redo that study with tools that they specifically favour (many of which violate the assumptions of our model) appears unreasonable and outside the scope of our paper.

4) The reviewer claims "the manuscript continues to overstate what its model can establish". However, we claim only that our model explains the observations, not that it is the sole explanation. Indeed, we have a full section in our Discussion section where we discuss alternative hypotheses.

In summary, we believe that Reviewer 1 is acting unreasonably to the extent that they are extreme

in their demands and resistance to views and methods that, to some degree, oppose their own. Whilst we appreciate that editors rely on unpaid reviewers who generally perform an invaluable role, Reviewer 1’s approach cannot be the best way to do science. It is highly likely that Reviewer 1 cannot be satisfied. We therefore request one of the following:

(a) Reviewers 1’s concerns are disregarded

(b) Reviewer 2 is consulted regarding the validity of Reviewer 1’s concerns or

(c) a further reviewer is asked to evaluate our manuscript. We are happy for this communication to be shared.

Thank you again for dealing with this matter.

Yours sincerely,

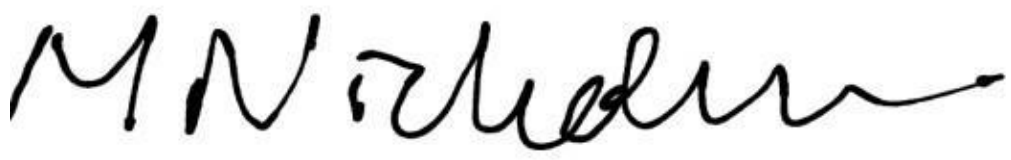

**Dr Michael Nicholson**
Chancellor’s Fellow
michael.nicholson@ed.ac.uk

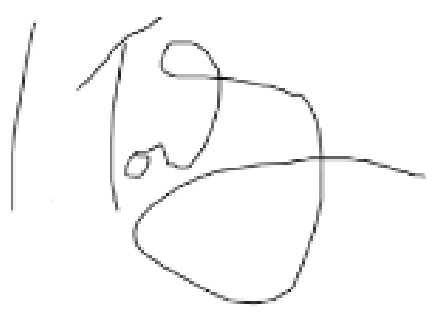

**Professor Ian Tomlinson FRS FMedSci**
Professor of Cancer Genetics
ian.tomlinson@oncology.ox.ac.uk
