# Supplementary material for: Hyper-mutational processes provide a head-start for weak cancer drivers: Explaining atypical KRAS variants
Source: PLoS Comput Biol. 2026 Oct 9;22(10):e1014858. doi: 10.1371/journal.pcbi.1014858 (PMC13674863; doi:10.1371/journal.pcbi.1014858)
Supplement: Supplementary file 5 [file pcbi.1014858.s002.docx]

S1 Fig: Contingency tables, odds ratios, and p-values (Fisher’s test) for different strong/weak criteria for *KRAS* driver variants in the cBioPortal data.
