# Supplementary material for: Hyper-mutational processes provide a head-start for weak cancer drivers: Explaining atypical KRAS variants
Source: PLoS Comput Biol. 2026 Oct 9;22(10):e1014858. doi: 10.1371/journal.pcbi.1014858 (PMC13674863; doi:10.1371/journal.pcbi.1014858)
Supplement: Supplementary file 7 [file pcbi.1014858.s004.docx]

S3 Fig: (left) Prior samples of the weak driver mutation rate in non-hypermutant used for the approximate Bayesian computation analysis; (right) Posterior samples of the weak driver mutation rate in non-hypermutant resulting from the approximate Bayesian computation analysis.
