# Supplementary material for: Hyper-mutational processes provide a head-start for weak cancer drivers: Explaining atypical KRAS variants
Source: PLoS Comput Biol. 2026 Oct 9;22(10):e1014858. doi: 10.1371/journal.pcbi.1014858 (PMC13674863; doi:10.1371/journal.pcbi.1014858)
Supplement: Supplementary file 8 [file pcbi.1014858.s008.docx]

We would like to thank the reviewers for their time, insights, and constructive feedback. We respond to their comments below and provide details on the changes we have made to the paper, which we believe have greatly improved our study.

To summarise, the main changes are:

- Restructuring of the discussion with emphasises on alternative hypotheses, including comments on immunological and epistatic mechanisms for explaining atypical *KRAS* variants.
- Additional analysis comparing proportion of tumours with weak/strong *KRAS* variants and simulations, which better informs on acceptable parameter regimes.
- Additional data analysis and simulations regarding *KRAS* variants in microsatellite unstable CRCs without *POLE* mutations.

Our point by point response can be found below.

**Referee #1 (Remarks to the Author):**

Reviewer #1: Review of “Hyper-mutational processes provide a head-start for weak cancer drivers, explaining atypical KRAS variants”

This manuscript shows that aberrant mutational processes alone can account for the detection of weak, rather than canonical, driver mutations. It demonstrates that simply increasing the mutation rate per cell division can lead to increased prevalence of weak drivers in high-TMB tumors due to the rapid occurrence of additional driver mutations—an effect that is enhanced by biases of mutations toward specific nucleotide sequence contexts. The manuscript quantifies the mutation bias toward varied KRAS drivers in both POLE-mutant and non-hypermutant colorectal cancers. It shows that the observed bias, coupled with the elevated mutation rate of POLE-mutant cancers, is sufficient to explain the appearance of atypical KRAS drivers in the hypermutant tumours. The manuscript concludes that differential selection between these cancer types need not be invoked to explain the variation in driver mutations, which may clarify the interplay of mutation and selection during the evolutionary dynamics of tumorigenesis.

We thank the reviewer for this succinct summary of our paper.

However, for it to contribute substantially to our understanding of the interplay of mutation and selection during the evolutionary dynamics of tumorigenesis, three major issues need to be addressed:

(1)—The scholarship of some components of the introduction could be improved/updated, and methodology associated with analysis and modeling potentially altered in consequence. Below are sentences extracted from the manuscript introduction, followed by relevant examples of research context in the literature that should be referenced either to support the point better, to add additional context, or to update the reference that was cited to support the point in the manuscript:

“cancer subtypes within a given tissue - where subtypes are delineated by measures as simple as mutation burden - have divergent driver variants”: e.g. [1–4]

“For example, in the 'hypermutant' class of colorectal cancers (CRCs), the most frequently mutated driver genes are ACRV2A, APC, and TGFBR2, which contrasts with non-hypermutant cancers where the classic triplet of APC, TP53, and KRAS are the most commonly mutated driver genes”: [5–7]

“For example, codon 12 mutations of the oncogene KRAS result in more potent biochemical activation of the K-ras protein, increased potential for oncogenesis, and elevated in-vitro growth rates, relative to other cancer-associated KRAS alleles”: [1,3,8–10]

“the exposure of a tumour to mutational processes is associated with the driver variants present in the cancer at sequencing“: [11–14]

“POLE-mutant CRCs … exhibit generally elevated mutation rates, that is a substantially increased number of mutations per cell division, resulting in orders of magnitude higher mutation burden than other cancers”: [15]

“The counter-argument made to the ‘mutational process’-centred explanation for weak drivers is that, as cancers develop over many years, and their clonal composition is determined by an optimising evolutionary process, cells containing sub-optimal driver variants should be displaced by fitter clones containing optimal drivers”: There is an alternate way to understand the pattern of weak drivers in high-TMB tumors, which is that weak drivers have antagonistic selective epistasis with strong drivers, preventing their subsequent fixation, and that oncogenesis by weak drivers requires higher underlying mutation to assemble sufficient oncogenicity to lead to a tumor. See major revision (3), below.

RESPONSE:

We acknowledge that in several places our scholarship could have been improved and thank the reviewer for bringing these references to our attention. We have added the main references suggested which we believe strengthen the points we are making with the highlighted sentences. While we appreciate the reviewer highlighting all of these references, so as to not dilute our points, we have not added all the references.

We respond to the reviewer’s remarks on alternative explanations in point 3 below.

ACTIONS:

- Included the suggested references which mostly strengthen our points, as well as additional references which improve scholarship.

(2)—With regard to their minimal mathematical model: mutation and selection is currently parameterized arbitrarily: that arbitrariness of the simulations is unjustified, as specific values (or at least specific ratios of values) of mutation rate and selection strength for each variant can be determined, based on tumor sequence data analysis. The manuscript model should be parameterized based on data-driven estimates of mutation rates and selection strengths, which can be obtained by publicly available methods applied to publicly available datasets [or extracted directly from published datasets; 16,17]. Such estimates may not be provided in the literature or by these tools _in the units desired for this model_. However, their ratios of mutation rate and of selection strength are independent of their unit of measurement in time / cancer stage. Application of extant tools to the cohort of POLE-mutant CRCs and the cohort of wildtype CRCs would provide

their relative mutation rates.

We agree with the reviewer that our simulations and model should be adequately parameterised. To this end, in our original supplementary file, we had two sections relevant to this, which we believe the reviewer might have missed. The first “Representative mutation spectra and *KRAS* variant rate” details how we use the PCAWG data to derive representative spectra and how we combine it with *KRAS* sequence contexts to estimate mutational bias to variants. The second section was titled “Mutation and selection parameter regime” where we collate parameter estimates in the literature, specifically relevant to the branching process model we employ.

We have moved those supplementary aspects relevant to data to the main text methods, which now contains details on representative spectra construction. To facilitate better exploration of the supplementary information file, we have now added a table of contents, as the “Mutation and selection parameter regime” was particularly buried under the mathematical derivations.

ACTIONS:

- Updated main text methods and added a table of contents to supplementary information.

(3)—The model assumes non-epistatic interactions among drivers. There is ample evidence not only of mutual exclusivity [too many references to list] but also of strong selective epistasis among drivers in cancer evolution [18–22], so results from any model assuming no selective epistasis are suspect. The analysis / modeling in this manuscript must account for the effects of epistasis, which is an alternate explanation for why weak drivers would not be replaced by strong drivers. Pairwise [17,18,23] and higher-order [24] epistatic effects can be quantified with publicly available methods applied to publicly available datasets.

RESPONSE:

We agree that weak drivers are likely to show antagonistic selective epistasis with strong drivers. This is in part why we specified that cells only acquire either weak or strong drivers, and this is now explicitly stated.

“As the vast majority of CRCs contain only one detectable *KRAS* variant (van Ginkel, Tomlinson, and Soriano 2023), and excessive oncogenic RAS signalling - potentially achievable through co-occurrence of both a weak and strong driver - may induce senescence [(Li et al. 2018)](https://paperpile.com/c/Mqk361/gGCD), we assume that cells only have either a weak or a strong driver, with each driver type arising only once.”

Thus in our model, we explicitly account for the reviewers comment that “ weak drivers have antagonistic selective epistasis with strong drivers”. However in our model this does not ‘prevent the strong driver’s “fixation” ‘ (~ reaching clonal dominance in our model, as there is no true fixation), because the strong driver may still arise from within the precursor population. This is the key difference between our model and Armitage-Doll (linear evolution) type models, where there can only be one genotype of the pre-cancerous cell(s) at any moment.

Similarly, while it is plausible that weak drivers work co-operatively, if those weak drivers are acquired during tumour growth - the focus of our model - this does not explain why a strong driver does not arise in the precursor population and eventually dominate the tumour. Indeed, if we amended our model so that tumours need to acquire two RAS drivers before a further critical driver event, this would make it even more unlikely to see weak *KRAS* variants in hypermutant CRCs. In [Woolley et al. 2025](https://eur02.safelinks.protection.outlook.com/?url=https%3A%2F%2Fpaperpile.com%2Fc%2FMqk361%2FNhsK&data=05%7C02%7C%7C38d7a832b3694ad5e13208de64aa13a2%7C2e9f06b016694589878910a06934dc61%7C0%7C0%7C639058878812019213%7CUnknown%7CTWFpbGZsb3d8eyJFbXB0eU1hcGkiOnRydWUsIlYiOiIwLjAuMDAwMCIsIlAiOiJXaW4zMiIsIkFOIjoiTWFpbCIsIldUIjoyfQ%3D%3D%7C0%7C%7C%7C&sdata=CpKaseGr31N54UQb%2B%2BGQoepYxAO%2F%2FzLQt8sHVMFCm8Y%3D&reserved=0) ( PMID: [39751654](https://pubmed.ncbi.nlm.nih.gov/39751654/)), we found that CRCs cells with atypical *BRAF* mutations tended to acquire additional, often weak mutations in other Ras pathway genes. However, despite that finding, the great majority (86.7%) of CRCs with atypical *KRAS* variants did not have further driver mutations in major Ras oncogenes, and those that did were not explained by hypermutation. Thus, we feel that amending our model will not improve its explanatory power for the particular question we consider, which is when and why clones with weaker mutations fail to be displaced by clones with stronger mutations.

To highlight the epistatic analysis of Wooley et al, and for readers who may have similar questions to the reviewer, we have added

“ One could hypothesise that multiple weak RAS drivers cumulatively initiate tumourigenesis - a path more accessible in hypermutants due to increased combinatorial opportunities. However, recent analyses [(Woolley et al. 2025)](https://paperpile.com/c/Mqk361/NhsK) found that the vast majority of CRCs with atypical *KRAS* variants did not have further driver mutations in major RAS oncogenes, and those that did were not explained by hypermutation.”

to the discussion section.

ACTIONS:

- Highlighted potential antagonistic epistatic effects of strong plus weak drivers when introducing the model.
- Highlighted recent analyses on co-occurrence of atypical KRAS drivers with alternative RAS drivers.

References cited

1. KRAS G12C-associated immunotherapy benefit in NSCLC is substantially mediated by tobacco-induced tumor mutation burden, not allele-specific effects. Lung Cancer. 2025;209: 108749.

2. Mandell JD, Fisk JN, Cyrenne E, Xu ML, Cannataro VL, Townsend JP. Not only mutations but also tumorigenesis can be substantially attributed to DNA damage from reactive oxygen species in RUNX1::RUNX1T1-fusion-positive acute myeloid leukemia. Leukemia. 2022;36: 2931–2933.

3. Tan C, Mandell JD, Dasari K, Cannataro VL, Alfaro-Murillo JA, Townsend JP. Heavy mutagenesis by tobacco leads to lung adenocarcinoma tumors with KRAS G12 mutations other than G12D, leading KRAS G12D tumors-on average-to exhibit a lower mutation burden. Lung Cancer. 2022;166: 265–269.

4. Viana-Errasti J, Marín R, García-Mulero S, Pons T, Terradas M, Capellá G, et al. Comparative Analysis of Somatic and Germline Polymerase Proofreading Deficiencies in Cancer: Molecular and Clinical Implications. Mod Pathol. 2025;38: 100843.

5. Wang J, Li R, He Y, Yi Y, Wu H, Liang Z. Next-generation sequencing reveals heterogeneous genetic alterations in key signaling pathways of mismatch repair deficient colorectal carcinomas. Mod Pathol. 2020;33: 2591–2601.

6. Lan Y-T, Chang S-C, Lin P-C, Lin C-H, Liang W-Y, Chen W-S, et al. High concordance of mutation patterns in 10 common mutated genes between tumor tissue and cell-free DNA in metastatic colorectal cancer. Am J Cancer Res. 2021;11: 2228–2237.

7. Huang W, Li W, Xu N, Li H, Zhang Z, Zhang X, et al. Differences in DNA damage repair gene mutations between left- and right-sided colorectal cancer. Cancer Med. 2023;12: 10187–10198.

8. Cannataro VL, Gaffney SG, Stender C, Zhao Z-M, Philips M, Greenstein AE, et al. Heterogeneity and mutation in KRAS and associated oncogenes: evaluating the potential for the evolution of resistance to targeting of KRAS G12C. Oncogene. 2018;37: 2444–2455.

9. Varshavi D, Varshavi D, McCarthy N, Veselkov K, Keun HC, Everett JR. Metabolic characterization of colorectal cancer cells harbouring different KRAS mutations in codon 12, 13, 61 and 146 using human SW48 isogenic cell lines. Metabolomics. 2020;16: 51.

10. Lavacchi D, Fancelli S, Roviello G, Castiglione F, Caliman E, Rossi G, et al. Mutations matter: An observational study of the prognostic and predictive value of KRAS mutations in metastatic colorectal cancer. Front Oncol. 2022;12: 1055019.

11. Fisk JN, Mahal AR, Dornburg A, Gaffney SG, Aneja S, Contessa JN, et al. Premetastatic shifts of endogenous and exogenous mutational processes support consolidative therapy in EGFR-driven lung adenocarcinoma. Cancer Lett. 2022;526: 346–351.

12. Poulos RC, Wong YT, Ryan R, Pang H, Wong JWH. Analysis of 7,815 cancer exomes reveals associations between mutational processes and somatic driver mutations. PLoS Genet. 2018;14: e1007779.

13. Wong JKL, Aichmüller C, Schulze M, Hlevnjak M, Elgaafary S, Lichter P, et al. Association of mutation signature effectuating processes with mutation hotspots in driver genes and non-coding regions. Nat Commun. 2022;13: 178.

14. Fang H, Barbour JA, Poulos RC, Katainen R, Aaltonen LA, Wong JWH. Mutational processes of distinct POLE exonuclease domain mutants drive an enrichment of a specific TP53 mutation in colorectal cancer. PLoS Genet. 2020;16: e1008572.

15. Forgó E, Gomez AJ, Steiner D, Zehnder J, Longacre TA. Morphological, immunophenotypical and molecular features of hypermutation in colorectal carcinomas with mutations in DNA polymerase ε (POLE). Histopathology. 2020;76: 366–374.

16. Cannataro VL, Gaffney SG, Townsend JP. Effect Sizes of Somatic Mutations in Cancer. J Natl Cancer Inst. 2018;110: 1171–1177.

17. Mandell JD, Cannataro VL, Townsend JP. Estimation of Neutral Mutation Rates and Quantification of Somatic Variant Selection Using cancereffectsizeR. Cancer Res. 2023;83: 500–505.

18. Iranzo J, Gruenhagen G, Calle-Espinosa J, Koonin EV. Pervasive conditional selection of driver mutations and modular epistasis networks in cancer. Cell Rep. 2022;40: 111272.

19. van de Haar J, Canisius S, Yu MK, Voest EE, Wessels LFA, Ideker T. Identifying Epistasis in Cancer Genomes: A Delicate Affair. Cell. 2019;177: 1375–1383.

20. Shen JP, Ideker T. Synthetic Lethal Networks for Precision Oncology: Promises and Pitfalls. J Mol Biol. 2018;430: 2900–2912.

21. Mina M, Iyer A, Ciriello G. Epistasis and evolutionary dependencies in human cancers. Curr Opin Genet Dev. 2022;77: 101989.

22. Dasari K, Somarelli JA, Kumar S, Townsend JP. The somatic molecular evolution of cancer: Mutation, selection, and epistasis. Prog Biophys Mol Biol. 2021;165: 56–65.

23. Iranzo J, Gruenhagen G, Calle-Espinosa J, Koonin EV. Protocol for comparing gene-level selection on coding mutations between two groups of samples with Coselens. STAR Protoc. 2023;4: 102117.

24. Alfaro-Murillo JA, Townsend JP. Pairwise and higher-order epistatic effects among somatic cancer mutations across oncogenesis. Math Biosci. 2023;366: 109091.

Reviewer #2: In this paper the authors explore the question of why POLE hypermutant colorectal cancers contain atypical KRAS driver mutations. Using a mathematical model and comparison with cancer genome sequencing data, they show that this can be explained by hypermutant tumours being more likely to acquire additional drivers in quick succession. This means that typical KRAS drivers that may be acquired in different subclones do not have sufficient time to outcompete the "weaker" drivers. Overall, the paper is well motivated, the mathematical model builds on classic branching process models of cancer evolution, it is well written and clear, and the comparison with CRC data is reasonable. This provides a useful new framework that demonstrates the interplay of selection and mutation on cancer driver landscapes. There are a few points that I feel could be added to strengthen the overall conclusions.

We thank the reviewer for the positive remarks and succinct summary.

Major items

1. In the final analysis related to Figure 6, I think it would aid the reader to report the observed proportion of weak versus strong drivers in POLE-mutant versus POLE-wildtype tumours. This would help interpret the results of the mathematical model and suggest plausible parameter regimes. Specifically, a figure or panel showing the "KRAS driver class" distribution from the actual data (rather than from the model predictions) could be added.

RESPONSE:

We thank the reviewer for this suggestion. The proportion of weak/strong drivers using the codon 12/13 criterion was actually shown as a motivating observation in Fig 1A. To re-emphasise these proportions we have added Fig 6D which shows the proportions versus simulations.

The reviewer makes an important point that the tumour proportion with weak drivers might be informative regarding parameter spaces explaining the data. To investigate this further we added an approximate Bayesian computation analysis (Fig 6. E-F), which showed that while univariate parameter estimates could not be obtained, the scaled selection difference can be well estimated. We further used these parameter estimates to (i) emphasise our key point that mutation rate plays a critical role in which drivers are detected (Fig 6G) and (ii) parameterise simulations exploring weak *KRAS* drivers in MSI tumours (see below).

ACTIONS:

- Further simulation based analysis resulted in new results (last two paragraphs of section “*KRAS* drivers in *POLE*-mutant cancers”) and new plots, Figs. 6D-G.

2. Mismatch repair deficient (MMRd) tumours are only briefly mentioned in the discussion. MMRd tumours represent an intermediate mutation rate between POLE-mutant and microsatellite stable tumours, and so one might expect some intermediate distribution of atypical KRAS variants. Did the authors explore whether MMRd tumours have atypical KRAS mutations? I recognize that the distribution of driver mutations in the MAPK/ERK pathway differs between MSS and MMRd tumours, but at least including some discussion of what the KRAS variant spectrum looks like in MMRd would be valuable and would make for a more complete study.

RESPONSE:

We thank the reviewer for this suggestion. In response we analysed MSI annotated CRCs from the 100,000 genomes project and in our original cBioPortal cohort, that did not have pathogenic *POLE* variants. Interestingly, we found that MSI tumours with mutant *KRAS* displayed an intermediate proportion of weak drivers when compared to non-hypermutants and *POLE*-mutant tumours (25% compared to 15% and 74%). To examine whether these values were consistent with our model and the parameters inferred from non-hypermutant and *POLE*-mutant tumours, we inferred the mutational bias expected to weak drivers under a MSI CRC mutational spectra and simulated tumour cohorts with 10X fold increase in SNV rate relative to non-hypermutants. The resulting analysis has been added as the final subsection of our Discussion “Mismatch repair deficient tumours“ with resulting new figure Fig. 6H.

As the reviewer points out MMRd tumours typically have different MAPK drivers, and often have different molecular evolutionary pathways. Thus, we have been careful to highlight these limitations in this new section.

ACTIONS:

- Additional data added, further data analysis on MSI tumours without *POLE* mutation, and further simulation based analysis resulting in a new results “Mismatch repair deficient tumours “ section and new plot, Fig. 6H.

3. One aspect not discussed is the possible effect of immune selection. POLE tumours respond exceptionally well to immunotherapy, presumably due to their extremely high neoantigen burden. Driver mutations themselves can be antigenic and there can be a trade-off between oncogenicity and antigenicity, best exemplified by TP53 mutations (PMID: 35545680). Something similar could be at play for KRAS drivers in CRC, and there are reports of the differential antigenicity of certain KRAS variants (PMID: 27959684). Therefore, it is plausible that high immunological activity in POLE tumours could also influence the selection landscape of KRAS drivers. While a full investigation may be beyond the scope of the paper, a few sentences addressing this possibility in the discussion would provide valuable additional context.

We thank the reviewer for pointing out this alternative hypothesis, and further references. We have added a discussion of this mechanism to our new “Alternative hypotheses” subsection of our discussion.

ACTIONS:

- Added paragraph on variable antigenicity of *KRAS* alleles in “Alternative hypotheses” subsection of Discussion.

Minor items

1. I did not see a code availability statement.

Code availability statement added.

2. Figure 6 needs panel labels added.

Corrected, thanks.

3. I think Figure 5A may be labelled incorrectly - the dark blue and light blue legend entries need to be swapped.

Corrected, thanks.

4. In the paragraph beginning "A curated list..." (related to Figure 5), results for all the different criteria for driver strength could be made more explicit, with the odds ratio and exact p-values reported for each classification scheme.

A supplementary figure with the contingency tables, as well as odds ratios and exact p-values is now provided as supplementary figure 1.

5. The discussion of results related to Figure 6 was unclear in certain sections. Specifically, in the paragraph beginning "We used the mathematical model...", the connection to Figure 6 should be made explicit. For example, for the sentence "Using the other classification criteria gave similar results," please clarify which results and which figure panel is being referenced.

We have clarified our discussion of the results. For the “other classification criteria” query we have added a supplementary figure 2 and referenced it at this point.
