# Supplementary material for: Hyper-mutational processes provide a head-start for weak cancer drivers: Explaining atypical KRAS variants
Source: PLoS Comput Biol. 2026 Oct 9;22(10):e1014858. doi: 10.1371/journal.pcbi.1014858 (PMC13674863; doi:10.1371/journal.pcbi.1014858)
Supplement: Supplementary file 9 [file pcbi.1014858.s010.docx]

We would like to thank reviewer 3 for their time and constructive feedback. We agree with all comments made by this reviewer and detail the changes made to the paper based on their feedback below.

We have also added the following sentence to the abstract to reflect the results on mismatch repair deficient tumours, motivated by reviewer 1 and added in the last review round:

*Furthermore, model predictions are consistent with the observed prevalence of atypical KRAS drivers observed in mismatch repair deficient colorectal cancer.*

**Referee #3 (Remarks to the Author):**

As requested, although I have studied the whole manuscript, I have focussed my review on considering Reviewer 1’s three outstanding concerns and the authors' responses to them. In summary, although I agree with Reviewer 1 that, as is almost always the case, the authors could do more to support their main claim, I don’t think the reviewer’s concerns merit major revisions. I suggest instead minor revisions to the Introduction and Discussion.

We thank the reviewer for this balanced assessment of our manuscript.

Point 1: Regarding “scholarship and framing”, it appears to me that all statements in the Introduction are supported by adequate references. Given that the reviewer neither identifies factual inaccuracies nor suggests additions to the text, I see no compelling reason to add further references.

We thank the reviewer for their favourable assessment of our scholarship and framing.

Point 2: Based on various relevant, good-quality studies, the authors have chosen to consider an extremely wide range of selection coeﬃcients and mutation rates that are broadly consistent with similar mathematical modelling studies. The reviewer would rather they take parameter values from alternative sources but provides no reason to believe that these would lead to substantially different results. In their cover letter, the authors make a reasonable argument that the alternative parameter values are unsuitable because they were inferred from a very different type of mathematical model. Moreover, I would argue that simplifying assumptions of both types of mathematical model – most notably the omission of spatial structure and immune responses – are likely to be much more influential than the parameter values within reasonable ranges. For instance, mutants with small selection coefficients spread much more slowly in standard spatial models of tumour evolution than in branching processes, and (effective) selective sweeps are much less probable in the former than in the latter. I suggest acknowledging these simplifying assumptions in the “Limitations” subsection of the Discussion.

We fully agree with the reviewers comments and have added the following paragraph to our Limitations subsection of the Discussion

*We have adopted the simplest, branching process model of competing clonal lineages, in part so that we can compare with evolutionary parameters obtained using a similar framework. Notably we have omitted both spatial structure and immune response in our model* [*(Noble et al. 2022; Lakatos et al. 2020)*](https://paperpile.com/c/0LWqfy/oMW0+gLi0)*. Both effects would alter the range of biological parameters consistent with differential KRAS alleles appearing in the non-hypermutant and hypermutant subtypes, as well as altering the likelihood of an effective selective sweep which our model assumes occurs after the third driver event occurs* [*(Stein et al. 2026)*](https://paperpile.com/c/0LWqfy/qBJO)*.*

Point 3: The manuscript’s main claim is that “aberrant mutational processes alone can account for the detection of weak, rather than canonical, driver mutations”. The reviewer doesn’t dispute that this claim holds for the considered mathematical model and parameter values. But they point out that epistatic interactions between driver mutations may also be sufficient to explain the observation, and they argue that, because the mathematical model omits such epistasis, the manuscript provides insufficient evidence that its main claim holds for actual tumours. Although I agree it would be interesting to explore an extended model including both factors, I don’t think this is essential for the current manuscript. Since epistasis is expected to favour weak drivers, it’s doubtful that adding epistasis to the model would qualitatively change the results (nor does the reviewer suggest it would do so). Nevertheless, it would be helpful to add a paragraph engaging with the reviewer’s epistatic hypothesis in the “Alternative hypotheses for atypical KRAS variants” subsection of the Discussion, and to mention epistasis as a plausible contributing factor in the Introduction. Currently the manuscript never once uses the word “epistasis”.

We agree that the with the reviewer and have added the following sentence in the introduction:

*Moreover, the excess mutations result in an abundance of mutated driver genes* [*(Cornish et al. 2024)*](https://paperpile.com/c/0LWqfy/fB0r)*, which potentially shifts the epistatic landscape to favour sub-optimal drivers.*

And the following paragraph in the “Alternative hypotheses for atypical KRAS variants” subsection of the Discussion:

*Generally, epistasis is a possible cause of the pervasiveness of weaker driver genes* [*(Castro-Giner et al. 2015)*](https://paperpile.com/c/0LWqfy/kW59)*. Numerous variants are likely acquired between the POLE and KRAS mutations, which could alter the sweet spot for oncogenic RAS signalling* [*(Li et al. 2018)*](https://paperpile.com/c/0LWqfy/BFXw) *such that canonical KRAS drivers are no longer maximally oncogenic. Indeed, inferred epistatic interactions vary by KRAS allele* [*(Cook et al. 2021)*](https://paperpile.com/c/0LWqfy/UF6H)*, thus POLE mutational processes could bias mutations towards genes that positively epistatically interact with atypical KRAS alleles and antagonistically with canonical alleles. However, this hypothesis is currently challenging to evaluate as the posited interactions are at the earliest stages of tumourigenesis, whereas the cancer genomic data informs on mutation patterns at sampling. One manifestation of this mechanism is multiple weak RAS pathway drivers cumulatively acting to initiate tumourigenesis. Casting doubt on the universality of the epistatic mechanism, recent analyses* [*(Woolley et al. 2025)*](https://eur02.safelinks.protection.outlook.com/?url=https%3A%2F%2Fpaperpile.com%2Fc%2FMqk361%2FNhsK&data=05%7C02%7C%7Cc62650c83d604bb9631008df0e607dc5%7C2e9f06b016694589878910a06934dc61%7C0%7C0%7C639245479966311829%7CUnknown%7CTWFpbGZsb3d8eyJFbXB0eU1hcGkiOnRydWUsIlYiOiIwLjAuMDAwMCIsIlAiOiJXaW4zMiIsIkFOIjoiTWFpbCIsIldUIjoyfQ%3D%3D%7C0%7C%7C%7C&sdata=A5Mk%2Bbmc%2BnVCXC3zml3vx%2FBjVrWkoDg1urDVRo7HyUE%3D&reserved=0) *found that the vast majority of CRCs with atypical KRAS variants did not have further driver mutations in major RAS oncogenes, and those that did were not explained by hypermutation.*
