# Supplementary material for: Hyper-mutational processes provide a head-start for weak cancer drivers: Explaining atypical KRAS variants
Source: PLoS Comput Biol. 2026 Oct 9;22(10):e1014858. doi: 10.1371/journal.pcbi.1014858 (PMC13674863; doi:10.1371/journal.pcbi.1014858)
Supplement: Peer review history

# Peer review: Decision Letter 0

**DOI:** 10.1371/journal.pcbi.1014858.r001

**Authors:** Zhaolei Zhang, Ivana Bozic

26 Nov 2025

PCOMPBIOL-D-25-02161

Hyper-mutational processes provide a head-start for weak cancer

drivers, explaining atypical KRAS variants

PLOS Computational Biology

Dear Dr. Nicholson,

Thank you for submitting your manuscript to PLOS Computational Biology. After careful consideration, we feel that it has merit but does not fully meet PLOS Computational Biology's publication criteria as it currently stands. Therefore, we invite you to submit a revised version of the manuscript that addresses the points raised during the review process.

Please submit your revised manuscript by January 21, 2026. If you will need more time than this to complete your revisions, please reply to this message or contact the journal office at ploscompbiol@plos.org. When you're ready to submit your revision, log on to https://www.editorialmanager.com/pcompbiol/ and select the 'Submissions Needing Revision' folder to locate your manuscript file.

Please include the following items when submitting your revised manuscript:

* A rebuttal letter that responds to each point raised by the editor and reviewer(s). You should upload this letter as a separate file labeled 'Response to Reviewers'. This file does not need to include responses to formatting updates and technical items listed in the 'Journal Requirements' section below.

* A marked-up copy of your manuscript that highlights changes made to the original version. You should upload this as a separate file labeled 'Revised Manuscript with Track Changes'.

* An unmarked version of your revised paper without tracked changes. You should upload this as a separate file labeled 'Manuscript'.

If you would like to make changes to your financial disclosure, competing interests statement, or data availability statement, please make these updates within the submission form at the time of resubmission. Guidelines for resubmitting your figure files are available below the reviewer comments at the end of this letter

We look forward to receiving your revised manuscript.

Kind regards,

Ivana Bozic

Academic Editor

PLOS Computational Biology

Zhaolei Zhang

Section Editor

PLOS Computational Biology

**Additional Editor Comments:**

Authors should carefully address all points raised by the reviewers. Particular attention should be given to questions regarding code availability, what findings result from application of the model to MMRd tumors, and parametrization of the model.

**Journal Requirements:**

If the reviewer comments include a recommendation to cite specific previously published works, please review and evaluate these publications to determine whether they are relevant and should be cited. There is no requirement to cite these works unless the editor has indicated otherwise.

1) Please ensure that the CRediT author contributions listed for every co-author are completed accurately and in full.

At this stage, the following Authors/Authors require contributions: Michael D Nicholson, and Ian Tomlinson. Please ensure that the full contributions of each author are acknowledged in the "Add/Edit/Remove Authors" section of our submission form.

The list of CRediT author contributions may be found here: https://journals.plos.org/ploscompbiol/s/authorship#loc-author-contributions

2) We ask that a manuscript source file is provided at Revision. Please upload your manuscript file as a .doc, .docx, .rtf or .tex. If you are providing a .tex file, please upload it under the item type u2018LaTeX Source Fileu2019 and leave your .pdf version as the item type u2018Manuscriptu2019.

3) Please provide an Author Summary. This should appear in your manuscript between the Abstract (if applicable) and the Introduction, and should be 150-200 words long. The aim should be to make your findings accessible to a wide audience that includes both scientists and non-scientists. Sample summaries can be found on our website under Submission Guidelines:

https://journals.plos.org/ploscompbiol/s/submission-guidelines#loc-parts-of-a-submission

4) Please upload all main figures as separate Figure files in .tif or .eps format. For more information about how to convert and format your figure files please see our guidelines:

https://journals.plos.org/ploscompbiol/s/figures

5) We have noticed that you have uploaded Supporting Information files, but you have not included a list of legends. Please add a full list of legends for your Supporting Information files after the references list.

6) Please amend your detailed Financial Disclosure statement. This is published with the article. It must therefore be completed in full sentences and contain the exact wording you wish to be published.

1) State the initials, alongside each funding source, of each author to receive each grant. For example: "This work was supported by the National Institutes of Health (####### to AM; ###### to CJ) and the National Science Foundation (###### to AM)."

2) State what role the funders took in the study. If the funders had no role in your study, please state: "The funders had no role in study design, data collection and analysis, decision to publish, or preparation of the manuscript."

3) If any authors received a salary from any of your funders, please state which authors and which funders..

If you did not receive any funding for this study, please simply state: u201cThe authors received no specific funding for this work.u201d

7) Kindly revise your competing statement in the online submission form to align with the journal's style guidelines: 'The authors declare that there are no competing interests.'

**Reviewers' comments:**

Reviewer's Responses to Questions

**Comments to the Authors:**

**Please note here if the review is uploaded as an attachment.**

Reviewer #1: Review of “Hyper-mutational processes provide a head-start for weak cancer drivers, explaining atypical KRAS variants”

This manuscript shows that aberrant mutational processes alone can account for the detection of weak, rather than canonical, driver mutations. It demonstrates that simply increasing the mutation rate per cell division can lead to increased prevalence of weak drivers in high-TMB tumors due to the rapid occurrence of additional driver mutations—an effect that is enhanced by biases of mutations toward specific nucleotide sequence contexts. The manuscript quantifies the mutation bias toward varied KRAS drivers in both POLE-mutant and non-hypermutant colorectal cancers. It shows that the observed bias, coupled with the elevated mutation rate of POLE-mutant cancers, is sufficient to explain the appearance of atypical KRAS drivers in the hypermutant tumours. The manuscript concludes that differential selection between these cancer types need not be invoked to explain the variation in driver mutations, which may clarify the interplay of mutation and selection during the evolutionary dynamics of tumorigenesis.

However, for it to contribute substantially to our understanding of the interplay of mutation and selection during the evolutionary dynamics of tumorigenesis, three major issues need to be addressed:

(1)—The scholarship of some components of the introduction could be improved/updated, and methodology associated with analysis and modeling potentially altered in consequence. Below are sentences extracted from the manuscript introduction, followed by relevant examples of research context in the literature that should be referenced either to support the point better, to add additional context, or to update the reference that was cited to support the point in the manuscript:

“cancer subtypes within a given tissue - where subtypes are delineated by measures as simple as mutation burden - have divergent driver variants”: e.g. [1–4]

“For example, in the 'hypermutant' class of colorectal cancers (CRCs), the most frequently mutated driver genes are ACRV2A, APC, and TGFBR2, which contrasts with non-hypermutant cancers where the classic triplet of APC, TP53, and KRAS are the most commonly mutated driver genes”: [5–7]

“For example, codon 12 mutations of the oncogene KRAS result in more potent biochemical activation of the K-ras protein, increased potential for oncogenesis, and elevated in-vitro growth rates, relative to other cancer-associated KRAS alleles”: [1,3,8–10]

“the exposure of a tumour to mutational processes is associated with the driver variants present in the cancer at sequencing“: [11–14]

“POLE-mutant CRCs … exhibit generally elevated mutation rates, that is a substantially increased number of mutations per cell division, resulting in orders of magnitude higher mutation burden than other cancers”: [15]

“The counter-argument made to the ‘mutational process’-centred explanation for weak drivers is that, as cancers develop over many years, and their clonal composition is determined by an optimising evolutionary process, cells containing sub-optimal driver variants should be displaced by fitter clones containing optimal drivers”: There is an alternate way to understand the pattern of weak drivers in high-TMB tumors, which is that weak drivers have antagonistic selective epistasis with strong drivers, preventing their subsequent fixation, and that oncogenesis by weak drivers requires higher underlying mutation to assemble sufficient oncogenicity to lead to a tumor. See major revision (3), below.

(2)—With regard to their minimal mathematical model: mutation and selection is currently parameterized arbitrarily: that arbitrariness of the simulations is unjustified, as specific values (or at least specific ratios of values) of mutation rate and selection strength for each variant can be determined, based on tumor sequence data analysis. The manuscript model should be parameterized based on data-driven estimates of mutation rates and selection strengths, which can be obtained by publicly available methods applied to publicly available datasets [or extracted directly from published datasets; 16,17]. Such estimates may not be provided in the literature or by these tools _in the units desired for this model_. However, their ratios of mutation rate and of selection strength are independent of their unit of measurement in time / cancer stage. Application of extant tools to the cohort of POLE-mutant CRCs and the cohort of wildtype CRCs would provide their relative mutation rates.

(3)—The model assumes non-epistatic interactions among drivers. There is ample evidence not only of mutual exclusivity [too many references to list] but also of strong selective epistasis among drivers in cancer evolution [18–22], so results from any model assuming no selective epistasis are suspect. The analysis / modeling in this manuscript must account for the effects of epistasis, which is an alternate explanation for why weak drivers would not be replaced by strong drivers. Pairwise [17,18,23] and higher-order [24] epistatic effects can be quantified with publicly available methods applied to publicly available datasets.

References cited

1. KRAS G12C-associated immunotherapy benefit in NSCLC is substantially mediated by tobacco-induced tumor mutation burden, not allele-specific effects. Lung Cancer. 2025;209: 108749.

2. Mandell JD, Fisk JN, Cyrenne E, Xu ML, Cannataro VL, Townsend JP. Not only mutations but also tumorigenesis can be substantially attributed to DNA damage from reactive oxygen species in RUNX1::RUNX1T1-fusion-positive acute myeloid leukemia. Leukemia. 2022;36: 2931–2933.

3. Tan C, Mandell JD, Dasari K, Cannataro VL, Alfaro-Murillo JA, Townsend JP. Heavy mutagenesis by tobacco leads to lung adenocarcinoma tumors with KRAS G12 mutations other than G12D, leading KRAS G12D tumors-on average-to exhibit a lower mutation burden. Lung Cancer. 2022;166: 265–269.

4. Viana-Errasti J, Marín R, García-Mulero S, Pons T, Terradas M, Capellá G, et al. Comparative Analysis of Somatic and Germline Polymerase Proofreading Deficiencies in Cancer: Molecular and Clinical Implications. Mod Pathol. 2025;38: 100843.

5. Wang J, Li R, He Y, Yi Y, Wu H, Liang Z. Next-generation sequencing reveals heterogeneous genetic alterations in key signaling pathways of mismatch repair deficient colorectal carcinomas. Mod Pathol. 2020;33: 2591–2601.

6. Lan Y-T, Chang S-C, Lin P-C, Lin C-H, Liang W-Y, Chen W-S, et al. High concordance of mutation patterns in 10 common mutated genes between tumor tissue and cell-free DNA in metastatic colorectal cancer. Am J Cancer Res. 2021;11: 2228–2237.

7. Huang W, Li W, Xu N, Li H, Zhang Z, Zhang X, et al. Differences in DNA damage repair gene mutations between left- and right-sided colorectal cancer. Cancer Med. 2023;12: 10187–10198.

8. Cannataro VL, Gaffney SG, Stender C, Zhao Z-M, Philips M, Greenstein AE, et al. Heterogeneity and mutation in KRAS and associated oncogenes: evaluating the potential for the evolution of resistance to targeting of KRAS G12C. Oncogene. 2018;37: 2444–2455.

9. Varshavi D, Varshavi D, McCarthy N, Veselkov K, Keun HC, Everett JR. Metabolic characterization of colorectal cancer cells harbouring different KRAS mutations in codon 12, 13, 61 and 146 using human SW48 isogenic cell lines. Metabolomics. 2020;16: 51.

10. Lavacchi D, Fancelli S, Roviello G, Castiglione F, Caliman E, Rossi G, et al. Mutations matter: An observational study of the prognostic and predictive value of KRAS mutations in metastatic colorectal cancer. Front Oncol. 2022;12: 1055019.

11. Fisk JN, Mahal AR, Dornburg A, Gaffney SG, Aneja S, Contessa JN, et al. Premetastatic shifts of endogenous and exogenous mutational processes support consolidative therapy in EGFR-driven lung adenocarcinoma. Cancer Lett. 2022;526: 346–351.

12. Poulos RC, Wong YT, Ryan R, Pang H, Wong JWH. Analysis of 7,815 cancer exomes reveals associations between mutational processes and somatic driver mutations. PLoS Genet. 2018;14: e1007779.

13. Wong JKL, Aichmüller C, Schulze M, Hlevnjak M, Elgaafary S, Lichter P, et al. Association of mutation signature effectuating processes with mutation hotspots in driver genes and non-coding regions. Nat Commun. 2022;13: 178.

14. Fang H, Barbour JA, Poulos RC, Katainen R, Aaltonen LA, Wong JWH. Mutational processes of distinct POLE exonuclease domain mutants drive an enrichment of a specific TP53 mutation in colorectal cancer. PLoS Genet. 2020;16: e1008572.

15. Forgó E, Gomez AJ, Steiner D, Zehnder J, Longacre TA. Morphological, immunophenotypical and molecular features of hypermutation in colorectal carcinomas with mutations in DNA polymerase ε (POLE). Histopathology. 2020;76: 366–374.

16. Cannataro VL, Gaffney SG, Townsend JP. Effect Sizes of Somatic Mutations in Cancer. J Natl Cancer Inst. 2018;110: 1171–1177.

17. Mandell JD, Cannataro VL, Townsend JP. Estimation of Neutral Mutation Rates and Quantification of Somatic Variant Selection Using cancereffectsizeR. Cancer Res. 2023;83: 500–505.

18. Iranzo J, Gruenhagen G, Calle-Espinosa J, Koonin EV. Pervasive conditional selection of driver mutations and modular epistasis networks in cancer. Cell Rep. 2022;40: 111272.

19. van de Haar J, Canisius S, Yu MK, Voest EE, Wessels LFA, Ideker T. Identifying Epistasis in Cancer Genomes: A Delicate Affair. Cell. 2019;177: 1375–1383.

20. Shen JP, Ideker T. Synthetic Lethal Networks for Precision Oncology: Promises and Pitfalls. J Mol Biol. 2018;430: 2900–2912.

21. Mina M, Iyer A, Ciriello G. Epistasis and evolutionary dependencies in human cancers. Curr Opin Genet Dev. 2022;77: 101989.

22. Dasari K, Somarelli JA, Kumar S, Townsend JP. The somatic molecular evolution of cancer: Mutation, selection, and epistasis. Prog Biophys Mol Biol. 2021;165: 56–65.

23. Iranzo J, Gruenhagen G, Calle-Espinosa J, Koonin EV. Protocol for comparing gene-level selection on coding mutations between two groups of samples with Coselens. STAR Protoc. 2023;4: 102117.

24. Alfaro-Murillo JA, Townsend JP. Pairwise and higher-order epistatic effects among somatic cancer mutations across oncogenesis. Math Biosci. 2023;366: 109091.

Reviewer #2: In this paper the authors explore the question of why POLE hypermutant colorectal cancers contain atypical KRAS driver mutations. Using a mathematical model and comparison with cancer genome sequencing data, they show that this can be explained by hypermutant tumours being more likely to acquire additional drivers in quick succession. This means that typical KRAS drivers that may be acquired in different subclones do not have sufficient time to outcompete the "weaker" drivers. Overall, the paper is well motivated, the mathematical model builds on classic branching process models of cancer evolution, it is well written and clear, and the comparison with CRC data is reasonable. This provides a useful new framework that demonstrates the interplay of selection and mutation on cancer driver landscapes. There are a few points that I feel could be added to strengthen the overall conclusions.

Major items

1. In the final analysis related to Figure 6, I think it would aid the reader to report the observed proportion of weak versus strong drivers in POLE-mutant versus POLE-wildtype tumours. This would help interpret the results of the mathematical model and suggest plausible parameter regimes. Specifically, a figure or panel showing the "KRAS driver class" distribution from the actual data (rather than from the model predictions) could be added.

2. Mismatch repair deficient (MMRd) tumours are only briefly mentioned in the discussion. MMRd tumours represent an intermediate mutation rate between POLE-mutant and microsatellite stable tumours, and so one might expect some intermediate distribution of atypical KRAS variants. Did the authors explore whether MMRd tumours have atypical KRAS mutations? I recognize that the distribution of driver mutations in the MAPK/ERK pathway differs between MSS and MMRd tumours, but at least including some discussion of what the KRAS variant spectrum looks like in MMRd would be valuable and would make for a more complete study.

3. One aspect not discussed is the possible effect of immune selection. POLE tumours respond exceptionally well to immunotherapy, presumably due to their extremely high neoantigen burden. Driver mutations themselves can be antigenic and there can be a trade-off between oncogenicity and antigenicity, best exemplified by TP53 mutations (PMID: 35545680). Something similar could be at play for KRAS drivers in CRC, and there are reports of the differential antigenicity of certain KRAS variants (PMID: 27959684). Therefore, it is plausible that high immunological activity in POLE tumours could also influence the selection landscape of KRAS drivers. While a full investigation may be beyond the scope of the paper, a few sentences addressing this possibility in the discussion would provide valuable additional context.

Minor items

1. I did not see a code availability statement.

2. Figure 6 needs panel labels added.

3. I think Figure 5A may be labelled incorrectly - the dark blue and light blue legend entries need to be swapped.

4. In the paragraph beginning "A curated list..." (related to Figure 5), results for all the different criteria for driver strength could be made more explicit, with the odds ratio and exact p-values reported for each classification scheme.

5. The discussion of results related to Figure 6 was unclear in certain sections. Specifically, in the paragraph beginning "We used the mathematical model...", the connection to Figure 6 should be made explicit. For example, for the sentence "Using the other classification criteria gave similar results," please clarify which results and which figure panel is being referenced.

**********

**Have the authors made all data and (if applicable) computational code underlying the findings in their manuscript fully available?**

The PLOS Data policy requires authors to make all data and code underlying the findings described in their manuscript fully available without restriction, with rare exception (please refer to the Data Availability Statement in the manuscript PDF file). The data and code should be provided as part of the manuscript or its supporting information, or deposited to a public repository. For example, in addition to summary statistics, the data points behind means, medians and variance measures should be available. If there are restrictions on publicly sharing data or code —e.g. participant privacy or use of data from a third party—those must be specified.

Reviewer #1: **No:**  They promise to make it available at publication. I recommend making it available to reviewers (at least), and preferably just make it open code. That way it can be checked and verified as complete and comprehensible and executable prior to publication.

Reviewer #2: **No:**  There was no link to the code. All data was publicly available data.

**********

PLOS authors have the option to publish the peer review history of their article (what does this mean?). If published, this will include your full peer review and any attached files.

If you choose “no”, your identity will remain anonymous but your review may still be made public.

**Do you want your identity to be public for this peer review?**  For information about this choice, including consent withdrawal, please see our Privacy Policy.

Reviewer #1: No

Reviewer #2: No

**Figure resubmission:**

While revising your submission, we strongly recommend that you use PLOS’s NAAS tool (https://ngplosjournals.pagemajik.ai/artanalysis) to test your figure files. NAAS can convert your figure files to the TIFF file type and meet basic requirements (such as print size, resolution), or provide you with a report on issues that do not meet our requirements and that NAAS cannot fix.

After uploading your figures to PLOS’s NAAS tool - https://ngplosjournals.pagemajik.ai/artanalysis, NAAS will process the files provided and display the results in the "Uploaded Files" section of the page as the processing is complete. If the uploaded figures meet our requirements (or NAAS is able to fix the files to meet our requirements), the figure will be marked as "fixed" above. If NAAS is unable to fix the files, a red "failed" label will appear above. When NAAS has confirmed that the figure files meet our requirements, please download the file via the download option, and include these NAAS processed figure files when submitting your revised manuscript.

**Reproducibility:**

To enhance the reproducibility of your results, we recommend that authors of applicable studies deposit laboratory protocols in protocols.io, where a protocol can be assigned its own identifier (DOI) such that it can be cited independently in the future. Additionally, PLOS ONE offers an option to publish peer-reviewed clinical study protocols. Read more information on sharing protocols at https://plos.org/protocols?utm_medium=editorial-email&utm_source=authorletters&utm_campaign=protocols

# Author comment: Author response to Decision Letter 1

**DOI:** 10.1371/journal.pcbi.1014858.r002

6 Feb 2026

Attachment Submitted filename: weakeakDrivers_plosCB_ReviewerResponse.docx

# Peer review: Decision Letter 1

**DOI:** 10.1371/journal.pcbi.1014858.r003

**Authors:** Zhaolei Zhang, Ivana Bozic

16 Mar 2026

PCOMPBIOL-D-25-02161R1

Hyper-mutational processes provide a head-start for weak cancer

drivers: explaining atypical KRAS variants

PLOS Computational Biology

Dear Dr. Nicholson,

Thank you for submitting your manuscript to PLOS Computational Biology. After careful consideration, we feel that it has merit but does not fully meet PLOS Computational Biology's publication criteria as it currently stands. Therefore, we invite you to submit a revised version of the manuscript that addresses the points raised during the review process.

Please submit your revised manuscript by May 12, 2026. If you will need more time than this to complete your revisions, please reply to this message or contact the journal office at ploscompbiol@plos.org. When you're ready to submit your revision, log on to https://www.editorialmanager.com/pcompbiol/ and select the 'Submissions Needing Revision' folder to locate your manuscript file.

Please include the following items when submitting your revised manuscript:

* A letter that responds to each point raised by the editor and reviewer(s). You should upload this letter as a separate file labeled 'Response to Reviewers'. This file does not need to include responses to formatting updates and technical items listed in the 'Journal Requirements' section below.

* A marked-up copy of your manuscript that highlights changes made to the original version. You should upload this as a separate file labeled 'Revised Manuscript with Track Changes'.

* An unmarked version of your revised paper without tracked changes. You should upload this as a separate file labeled 'Manuscript'.

If you would like to make changes to your financial disclosure, competing interests statement, or data availability statement, please make these updates within the submission form at the time of resubmission. Guidelines for resubmitting your figure files are available below the reviewer comments at the end of this letter

We look forward to receiving your revised manuscript.

Kind regards,

Ivana Bozic

Academic Editor

PLOS Computational Biology

Zhaolei Zhang

Section Editor

PLOS Computational Biology

**Additional Editor Comments:**

Reviewer 2 is satisfied with the revision. However, before the paper can be considered for publication, the concerns of Reviewer 1 need to be addressed. In particular, the issues Reviewer 1 raised need to be engaged with thoroughly and new analyses that were suggested should be conducted.

**Reviewers' comments:**

Reviewer's Responses to Questions

**Comments to the Authors:**

**Please note here if the review is uploaded as an attachment.**

Reviewer #1: Thank you for the opportunity to review the revised manuscript and the response to peer review. The revision includes some useful changes, but the central concerns previously raised have not been resolved, and the manuscript still does not reach the level of rigor required for publication. The recommendation therefore remains rejection.

The original review identified three major issues that required substantive resolution for the manuscript to merit publication:

(1) The scholarship in parts of the Introduction required updating, with implications for framing and potentially for methodology.

(2) The mathematical model was parameterized in an unjustifiably crude fashion, despite the availability of data-driven estimates of mutation rates and selection coefficients for specific driver variants.

(3) The model assumes non-epistatic interactions among drivers, despite extensive evidence that selective epistasis is pervasive in cancer evolution and directly relevant to the phenomenon under study.

After consideration of the revision and the response to peer review, point (1) appears to have been addressed only partially, whereas points (2) and (3) have not been addressed in substance.

1. Scholarship and framing remain incompletely updated

The response to peer review indicates that “the main references suggested” were incorporated, but not all, “so as to not dilute our points.” That is not a satisfactory resolution. The omitted references were not peripheral; they were directly relevant to the manuscript’s central claims and were suggested precisely because they would strengthen the cogency, saliency, and modernity of the argument rather than distract from it.

This point is not simply a matter of citation density. The omitted literature bears directly on interpretation of the observed patterns, on what classes of models are appropriate, and on what degree of modeling and parameterization of this problem has already been done. Updating the scholarship was necessary not merely for completeness, but because the field has advanced materially beyond the framing adopted in the manuscript. The revision improves the Introduction to some extent, but it still does not adequately reflect the current state of knowledge.

2. The critique of model parameterization has not been answered

The response to peer review does not address the actual substance of the concern about parameterization. The issue was never whether some data had been used. The issue was whether the crudity of the parameterization is defensible in light of current knowledge. It is not.

The manuscript continues to rely on a coarse, old-style assignment of a very few of categories of mutation rate and selection strength, informed by approximate literature values and strong simplifying assumptions. That approach is not adequate here. Publicly available datasets and methods now permit estimation of mutation rates and selection coefficients—or at minimum their relative magnitudes—for specific driver variants and sites, for all the driver variants and sites identified. Even if the units of those estimates differ from those used in the branching-process framework, the relevant ratios of mutation rates and selective effects are still derivable in a data-driven manner and can be used to constrain or calibrate the model.

That work to bring to bear substantial advances in our understanding to date, has not been done. I appreciate the movement of key sections from the Supplement to the Methods and the addition of a table of contents to the Supplement. However, these changes improve accessibility but do not improve methodological rigor. The issue is not where the parameterization is described; the issue is that the parameterization itself remains incredibly crude compared to contemporary empirical estimates.

Because the manuscript advances a quantitative claim—namely, that mutational processes alone are sufficient to explain the prevalence of atypical KRAS variants—the burden is on the analysis to show that this conclusion is robust under realistic, data-derived parameterization. That burden has not been met.

3. The epistasis critique has likewise not been answered

The response to the concern regarding epistasis is also not satisfactory. The manuscript’s central explanatory claim is that elevated mutation and mutational bias can account for the observed pattern without invoking differential selection. Yet the model continues to exclude the very class of selective interactions most relevant to that inference: epistatic interactions among driver mutations.

A simpler model can be justified if the simplification preserves the logic of the inference. Here, it does not. To be more explicit: the response to peer review argues that prohibiting cells from carrying both a weak and a strong KRAS driver “explicitly accounts” for antagonistic epistasis. That is not persuasive. Such a restriction is extremely crude and categorical rather than specific to every driver (as is parameterizable now), and is not a meaningful treatment of the broader problem of selective epistasis in cancer evolution. It is a narrow modeling constraint that excludes one particular state. It does not capture the empirically documented network of antagonistic and synergistic interactions among drivers that shapes evolutionary accessibility, clonal competition, and persistence of genotypes during tumor evolution. In particular, it does not address the possibility—raised previously—that epistatic structure itself may explain why weak drivers arise and persist in certain contexts without being displaced by canonical stronger drivers.

This point is central rather than peripheral. If strong selective epistasis can itself generate the qualitative pattern that the manuscript seeks to explain, then a model that excludes such interactions cannot support the claim that mutation-centered mechanisms are sufficient. At most, the present model shows that one highly simplified, non-epistatic system can generate a pattern resembling the data. That is a substantially weaker conclusion than the manuscript advances, especially since it is at this point well-established that cancer evolution is not a non-epistatic system.

The newly added discussion of the infrequency of multiple weak RAS-pathway drivers in one dataset does not resolve this issue. Lack of frequent co-occurrence within a limited gene set is not equivalent to showing that epistasis is unimportant, and in fact is evidence of antagonistic epistasis. Nor does mention of a recent related analysis substitute for explicitly incorporating epistatic structure into the modeling or testing whether the principal conclusions are robust to empirically informed epistatic effects.

Overall assessment

In its present form, the manuscript continues to overstate what its model can establish. The revision does not convincingly show that atypical KRAS variants in hypermutant colorectal cancers can be explained by mutational processes alone in a manner that is robust to contemporary empirical estimates of mutation and selection or to the well-established epistatic structure of cancer-driver evolution. The model remains too arbitrarily parameterized, too biologically simplified in precisely the dimension most relevant to the manuscript’s core claim, and too weakly integrated with the current literature to support the strength of its conclusions.

For these reasons, the manuscript does not appear suitable for publication.

Reviewer #2: The authors have addressed all of my concerns thoroughly and I am satisfied with their revisions. I particularly appreciated the addition of the Approximate Bayesian Computation analysis, which went above and beyond what was requested and provides a welcome connection between the mathematical framework and the data. Congratulations on an elegant and well-executed study. One minor remaining point: in the new paragraph on immunogenicity in the discussion, the references appear to need swapping.

**********

**Have the authors made all data and (if applicable) computational code underlying the findings in their manuscript fully available?**

The PLOS Data policy requires authors to make all data and code underlying the findings described in their manuscript fully available without restriction, with rare exception (please refer to the Data Availability Statement in the manuscript PDF file). The data and code should be provided as part of the manuscript or its supporting information, or deposited to a public repository. For example, in addition to summary statistics, the data points behind means, medians and variance measures should be available. If there are restrictions on publicly sharing data or code —e.g. participant privacy or use of data from a third party—those must be specified.

Reviewer #1: Yes

Reviewer #2: Yes

**********

PLOS authors have the option to publish the peer review history of their article (what does this mean?). If published, this will include your full peer review and any attached files.

If you choose “no”, your identity will remain anonymous but your review may still be made public.

**Do you want your identity to be public for this peer review?**  For information about this choice, including consent withdrawal, please see our Privacy Policy.

Reviewer #1: No

Reviewer #2: No

[NOTE: If reviewer comments were submitted as an attachment file, they will be attached to this email and accessible via the submission site. Please log into your account, locate the manuscript record, and check for the action link "View Attachments". If this link does not appear, there are no attachment files.]

**Figure resubmission:**

While revising your submission, we strongly recommend that you use PLOS’s NAAS tool (https://ngplosjournals.pagemajik.ai/artanalysis) to test your figure files. NAAS can convert your figure files to the TIFF file type and meet basic requirements (such as print size, resolution), or provide you with a report on issues that do not meet our requirements and that NAAS cannot fix.

After uploading your figures to PLOS’s NAAS tool - https://ngplosjournals.pagemajik.ai/artanalysis, NAAS will process the files provided and display the results in the "Uploaded Files" section of the page as the processing is complete. If the uploaded figures meet our requirements (or NAAS is able to fix the files to meet our requirements), the figure will be marked as "fixed" above. If NAAS is unable to fix the files, a red "failed" label will appear above. When NAAS has confirmed that the figure files meet our requirements, please download the file via the download option, and include these NAAS processed figure files when submitting your revised manuscript.

**Reproducibility:**

To enhance the reproducibility of your results, we recommend that authors of applicable studies deposit laboratory protocols in protocols.io, where a protocol can be assigned its own identifier (DOI) such that it can be cited independently in the future. Additionally, PLOS ONE offers an option to publish peer-reviewed clinical study protocols. Read more information on sharing protocols at https://plos.org/protocols?utm_medium=editorial-email&utm_source=authorletters&utm_campaign=protocols

# Author comment: Author response to Decision Letter 2

**DOI:** 10.1371/journal.pcbi.1014858.r004

16 Apr 2026

Attachment Submitted filename: weakDriversPlosCbAppealLetter (2).pdf

# Peer review: Decision Letter 2

**DOI:** 10.1371/journal.pcbi.1014858.r005

**Authors:** Zhaolei Zhang, Ivana Bozic

5 Jun 2026

PCOMPBIOL-D-25-02161R2

Hyper-mutational processes provide a head-start for weak cancer

drivers: explaining atypical KRAS variants

PLOS Computational Biology

Dear Dr. Nicholson,

Thank you for submitting your manuscript to PLOS Computational Biology. After careful consideration, we feel that it has merit but does not fully meet PLOS Computational Biology's publication criteria as it currently stands. Therefore, we invite you to submit a revised version of the manuscript that addresses the points raised during the review process.

Please submit your revised manuscript by July 3, 2026. If you will need more time than this to complete your revisions, please reply to this message or contact the journal office at ploscompbiol@plos.org. When you're ready to submit your revision, log on to https://www.editorialmanager.com/pcompbiol/ and select the 'Submissions Needing Revision' folder to locate your manuscript file.

Please include the following items when submitting your revised manuscript:

* A letter that responds to each point raised by the editor and reviewer(s). You should upload this letter as a separate file labeled 'Response to Reviewers'. This file does not need to include responses to formatting updates and technical items listed in the 'Journal Requirements' section below.

* A marked-up copy of your manuscript that highlights changes made to the original version. You should upload this as a separate file labeled 'Revised Manuscript with Track Changes'.

* An unmarked version of your revised paper without tracked changes. You should upload this as a separate file labeled 'Manuscript'.

If you would like to make changes to your financial disclosure, competing interests statement, or data availability statement, please make these updates within the submission form at the time of resubmission. Guidelines for resubmitting your figure files are available below the reviewer comments at the end of this letter.

As the corresponding author, your ORCID iD is verified in the submission system and will appear in the published article. PLOS supports the use of ORCID, and we encourage all coauthors to register for an ORCID iD and use it as well. Please encourage your coauthors to verify their ORCID iD within the submission system before final acceptance, as unverified ORCID iDs will not appear in the published article. Only the individual author can complete the verification step; PLOS staff cannot verify ORCID iDs on behalf of authors.

We look forward to receiving your revised manuscript.

Kind regards,

Ivana Bozic

Academic Editor

PLOS Computational Biology

Zhaolei Zhang

Section Editor

PLOS Computational Biology

**Additional Editor Comments:**

Please address all comments and make all changes suggested by Reviewer 3.

**Reviewers' comments:**

Reviewer's Responses to Questions

**Comments to the Authors:**

**Please note here if the review is uploaded as an attachment.**

Reviewer #3: As requested, although I have studied the whole manuscript, I have focussed my review on considering Reviewer 1’s three outstanding concerns and the authors' responses to them. In summary, although I agree with Reviewer 1 that, as is almost always the case, the authors could do more to support their main claim, I don’t think the reviewer’s concerns merit major revisions. I suggest instead minor revisions to the Introduction and Discussion.

Point 1: Regarding “scholarship and framing”, it appears to me that all statements in the Introduction are supported by adequate references. Given that the reviewer neither identifies factual inaccuracies nor suggests additions to the text, I see no compelling reason to add further references.

Point 2: Based on various relevant, good-quality studies, the authors have chosen to consider an extremely wide range of selection coeﬃcients and mutation rates that are broadly consistent with similar mathematical modelling studies. The reviewer would rather they take parameter values from alternative sources but provides no reason to believe that these would lead to substantially different results. In their cover letter, the authors make a reasonable argument that the alternative parameter values are unsuitable because they were inferred from a very different type of mathematical model. Moreover, I would argue that simplifying assumptions of both types of mathematical model – most notably the omission of spatial structure and immune responses – are likely to be much more influential than the parameter values within reasonable ranges. For instance, mutants with small selection coefficients spread much more slowly in standard spatial models of tumour evolution than in branching processes, and (effective) selective sweeps are much less probable in the former than in the latter. I suggest acknowledging these simplifying assumptions in the “Limitations” subsection of the Discussion.

Point 3: The manuscript’s main claim is that “aberrant mutational processes alone can account for the detection of weak, rather than canonical, driver mutations”. The reviewer doesn’t dispute that this claim holds for the considered mathematical model and parameter values. But they point out that epistatic interactions between driver mutations may also be sufficient to explain the observation, and they argue that, because the mathematical model omits such epistasis, the manuscript provides insufficient evidence that its main claim holds for actual tumours. Although I agree it would be interesting to explore an extended model including both factors, I don’t think this is essential for the current manuscript. Since epistasis is expected to favour weak drivers, it’s doubtful that adding epistasis to the model would qualitatively change the results (nor does the reviewer suggest it would do so). Nevertheless, it would be helpful to add a paragraph engaging with the reviewer’s epistatic hypothesis in the “Alternative hypotheses for atypical KRAS variants” subsection of the Discussion, and to mention epistasis as a plausible contributing factor in the Introduction. Currently the manuscript never once uses the word “epistasis”.

**********

**Have the authors made all data and (if applicable) computational code underlying the findings in their manuscript fully available?**

The PLOS Data policy requires authors to make all data and code underlying the findings described in their manuscript fully available without restriction, with rare exception (please refer to the Data Availability Statement in the manuscript PDF file). The data and code should be provided as part of the manuscript or its supporting information, or deposited to a public repository. For example, in addition to summary statistics, the data points behind means, medians and variance measures should be available. If there are restrictions on publicly sharing data or code —e.g. participant privacy or use of data from a third party—those must be specified.

Reviewer #3: Yes

**********

PLOS authors have the option to publish the peer review history of their article (what does this mean?). If published, this will include your full peer review and any attached files.

If you choose “no”, your identity will remain anonymous but your review may still be made public.

**Do you want your identity to be public for this peer review?**  For information about this choice, including consent withdrawal, please see our Privacy Policy.

Reviewer #3: No

[NOTE: If reviewer comments were submitted as an attachment file, they will be attached to this email and accessible via the submission site. Please log into your account, locate the manuscript record, and check for the action link "View Attachments". If this link does not appear, there are no attachment files.]

**Figure resubmission:**

While revising your submission, we strongly recommend that you use PLOS’s NAAS tool (https://ngplosjournals.pagemajik.ai/artanalysis) to test your figure files. NAAS can convert your figure files to the TIFF file type and meet basic requirements (such as print size, resolution), or provide you with a report on issues that do not meet our requirements and that NAAS cannot fix.

After uploading your figures to PLOS’s NAAS tool - https://ngplosjournals.pagemajik.ai/artanalysis, NAAS will process the files provided and display the results in the "Uploaded Files" section of the page as the processing is complete. If the uploaded figures meet our requirements (or NAAS is able to fix the files to meet our requirements), the figure will be marked as "fixed" above. If NAAS is unable to fix the files, a red "failed" label will appear above. When NAAS has confirmed that the figure files meet our requirements, please download the file via the download option, and include these NAAS processed figure files when submitting your revised manuscript.

**Reproducibility:**

To enhance the reproducibility of your results, we recommend that authors of applicable studies deposit laboratory protocols in protocols.io, where a protocol can be assigned its own identifier (DOI) such that it can be cited independently in the future. Additionally, PLOS ONE offers an option to publish peer-reviewed clinical study protocols. Read more information on sharing protocols at https://plos.org/protocols?utm_medium=editorial-email&utm_source=authorletters&utm_campaign=protocols

# Author comment: Author response to Decision Letter 3

**DOI:** 10.1371/journal.pcbi.1014858.r006

22 Sep 2026

Attachment Submitted filename: weakDrivers_plosCB_ReviewerResponse_Round2.docx

# Editor report: Decision Letter 3

**DOI:** 10.1371/journal.pcbi.1014858.r007

**Authors:** Zhaolei Zhang, Ivana Bozic

27 Sep 2026

Dear Dr. Nicholson,

We are pleased to inform you that your manuscript 'Hyper-mutational processes provide a head-start for weak cancer

drivers: explaining atypical KRAS variants' has been provisionally accepted for publication in PLOS Computational Biology.

Before your manuscript can be formally accepted you will need to complete some formatting changes, which you will receive in a follow up email. A member of our team will be in touch with a set of requests.

Please note that your manuscript will not be scheduled for publication until you have made the required changes, so a swift response is appreciated.

IMPORTANT: The editorial review process is now complete. PLOS will only permit corrections to spelling, formatting or significant scientific errors from this point onwards. Requests for major changes, or any which affect the scientific understanding of your work, will cause delays to the publication date of your manuscript.

Should you, your institution's press office or the journal office choose to press release your paper, you will automatically be opted out of early publication. We ask that you notify us now if you or your institution is planning to press release the article. All press must be co-ordinated with PLOS.

Thank you again for supporting Open Access publishing; we are looking forward to publishing your work in PLOS Computational Biology.

Best regards,

Ivana Bozic

Academic Editor

PLOS Computational Biology

Zhaolei Zhang

Section Editor

PLOS Computational Biology

***********************************************************

# Editor report: Acceptance letter

**DOI:** 10.1371/journal.pcbi.1014858.r008

**Authors:** Zhaolei Zhang, Ivana Bozic

PCOMPBIOL-D-25-02161R3

Hyper-mutational processes provide a head-start for weak cancer

drivers: explaining atypical KRAS variants

Dear Dr Nicholson,

I am pleased to inform you that your manuscript has been formally accepted for publication in PLOS Computational Biology. Your manuscript is now with our production department and you will be notified of the publication date in due course.

The corresponding author will soon be receiving a typeset proof for review, to ensure errors have not been introduced during production. Please review the PDF proof of your manuscript carefully, as this is the last chance to correct any errors. Please note that major changes, or those which affect the scientific understanding of the work, will likely cause delays to the publication date of your manuscript.

Soon after your final files are uploaded, unless you have opted out, the early version of your manuscript will be published online. The date of the early version will be your article's publication date. The final article will be published to the same URL, and all versions of the paper will be accessible to readers.

For Research, Software, and Methods articles, you will receive an invoice from PLOS for your publication fee after your manuscript has reached the completed accept phase. If you receive an email requesting payment before acceptance or for any other service, this may be a phishing scheme. Learn how to identify phishing emails and protect your accounts at https://explore.plos.org/phishing.

Thank you again for supporting PLOS Computational Biology and open-access publishing. We are looking forward to publishing your work!

With kind regards,

Janani Seenivasan

PLOS Computational Biology | Carlyle House, Carlyle Road, Cambridge CB4 3DN | United Kingdom ploscompbiol@plos.org | Phone +44 (0) 1223-442824 | ploscompbiol.org | @PLOSCompBiol
